# Elevating Healthcare: Rapid Literature Review on Drone Applications for Streamlining Disaster Management and Prehospital Care in Saudi Arabia

**DOI:** 10.3390/healthcare11111575

**Published:** 2023-05-27

**Authors:** Ahmed M. Al-Wathinani, Mohammad A. Alhallaf, Marta Borowska-Stefańska, Szymon Wiśniewski, Mohammed Ali Salem Sultan, Omar Y. Samman, Abdullah M. Alobaid, Saqer M. Althunayyan, Krzysztof Goniewicz

**Affiliations:** 1 Department of Emergency Medical Services, Prince Sultan Bin Abdulaziz College for Emergency Medical Services, King Saud University, Riyadh 11362, Saudi Arabia; malhallaf@ksu.edu.sa; 2 Institute of the Built Environment and Spatial Policy, University of Lodz, 90-142 Lodz, Poland; marta.borowska.stefanska@geo.uni.lodz.pl (M.B.-S.); szymon.wisniewski@geo.uni.lodz.pl (S.W.); 3 Healthcare Transformation, Model of Care, Regional Health Directorate, Najran 66255, Saudi Arabia; mohdsultan01@hotmail.com; 4 Institute of Health and Care Sciences, Sahlgrenska Academy, Gothenburg University, 40530 Gothenburg, Sweden; 5 Ibn Sina National College for Medical Studies, Jeddah 22421, Saudi Arabia; 6 Department of Trauma and Accident, Prince Sultan Bin Abdulaziz College, King Saud University, Riyadh 11362, Saudi Arabia; abdmalobaid@ksu.edu.sa (A.M.A.); salthunayyan@ksu.edu.sa (S.M.A.); 7 Department of Security Studies, Polish Air Force University, 08-521 Dęblin, Poland; k.goniewicz@law.mil.pl

**Keywords:** drone technology, UAV, health sector, disaster response, prehospital care, emergency medical services

## Abstract

Saudi Arabia’s health sector faces pressing challenges in disaster and prehospital care delivery, such as prolonged response times, limited access to remote areas, and strained medical resources. Integrating drone technology has emerged as an innovative approach to address these challenges and revolutionize healthcare delivery. Drones can significantly enhance response times, increase access to underserved areas, and reduce the burden on existing medical infrastructure. A detailed analysis of global case studies demonstrates the successful use of drones in healthcare delivery, emphasizing the importance of regulatory frameworks and public–private partnerships. These examples provide valuable insights into Saudi Arabia’s health sector transformation. The potential benefits of integrating drone technology include improved patient outcomes, increased efficiency, and cost savings. To ensure the successful implementation of this transformative approach, it is crucial to establish clear regulatory guidelines, invest in research and development, and foster collaboration between the government, private sector, and healthcare stakeholders. The aim of this study is to explore the potential of drone technology in transforming healthcare delivery in Saudi Arabia, particularly within disaster response and prehospital care services.

## 1. Introduction

Drones, or unmanned aerial vehicles (UAVs), are gaining momentum as transformative technology in the realm of disaster medicine and prehospital care delivery [1]. Their multifaceted capabilities, ranging from aerial surveillance to medical supply delivery, are redefining the paradigms of emergency response in disaster-stricken regions [1,2].

Disasters, both natural and manmade, often entail complex situations that require swift and efficient management to mitigate human suffering. For instance, floods and earthquakes can lead to significant casualties and damage to infrastructure, thereby impeding the traditional means of emergency response. Drones, with their advanced technological capabilities, have shown promise in these challenging environments [3,4,5,6].

In search and rescue operations, drones can play a pivotal role in identifying and locating individuals trapped in disaster-stricken areas. Advanced drones are equipped with thermal imaging and infrared sensors that can detect heat signatures from human bodies, even under debris or in low-light conditions, thereby aiding the identification of trapped or injured individuals during earthquakes or building collapses [7,8]. This technology significantly increases the efficiency and safety of search and rescue operations, preventing additional human exposure to hazardous conditions.

Saudi Arabia, given its geographical expanse and varied topography, is prone to several types of disasters. These include frequent sandstorms, occasional floods, and earthquakes, particularly in the western and southwestern regions [9]. The country’s disaster management strategy has increasingly recognized the potential of drones in bolstering its emergency response capabilities [10].

One pioneering initiative in this domain is Medidrone, a unique system in Saudi Arabia developed to harness the potential of drones in healthcare delivery [11]. Medidrone aims to provide rapid emergency services to patients in remote or hard-to-reach areas. It encompasses a drone equipped with essential medical equipment, a mobile application for streamlined communication, and a web-based portal for real-time data collection and patient monitoring [11].

The drone, central to the Medidrone system, is capable of carrying medical equipment, including defibrillators, oxygen tanks, and medications, to locations where timely medical intervention is critical. Notably, the drone incorporates temperature control mechanisms to maintain optimal conditions for temperature-sensitive medical supplies, such as certain medications and blood products [12,13].

The mobile application serves as an interface for healthcare providers to request drone services, while the web-based portal enables real-time tracking of the drone’s location and patient condition, facilitating timely intervention. The Medidrone system has successfully been deployed in several emergency situations in Saudi Arabia, such as transporting medical supplies during floods and delivering emergency medical care to patients in remote areas [11].

However, it is important to note that drones are not the panacea for all healthcare delivery challenges. While they can overcome geographical constraints and provide rapid response, there are regulatory, safety, and technological hurdles to their wider adoption [14]. Nevertheless, the potential of drones in transforming healthcare delivery, particularly in disaster management and prehospital care, is vast and warrants further exploration.

This article delves into the integration of drones in healthcare delivery in Saudi Arabia. It discusses their potential to augment disaster and prehospital care delivery, focusing on search and rescue operations, medical supply delivery, and remote monitoring of disaster-affected areas. It also touches upon regulatory considerations, technological advancements, and future directions for drone usage in healthcare delivery. Through this discourse, the article aims to highlight the transformative potential of drones in healthcare delivery in Saudi Arabia and beyond.

This manuscript follows a systematic structure. Section 2 delineates the materials and methods, detailing our search strategy and inclusion and exclusion criteria, as well as the data extraction and analysis processes. Section 3 elaborates on the major themes found in the literature, particularly focusing on the potential roles of drones in disaster response and prehospital care, while also pointing towards future research directions.

Section 4 presents a thorough analysis of the results and their implications, enhancing the overall understanding of drone applications in healthcare. Section 5 addresses the study’s limitations, necessary for accurate interpretation of the findings and for guiding future research. Lastly, Section 6 concludes the paper, summarizing the key findings and underlining the transformative potential of drones in healthcare, alongside the challenges that need to be overcome for wider adoption. The aim of this structured discussion is to provide a comprehensive overview of the topic and inspire further investigation in this important area of healthcare innovation.

## 2. Materials and Methods

In this study, we employed a rapid review methodology, an approach increasingly recognized as a rigorous and efficient method for quickly synthesizing research evidence in a time-sensitive context [15]. This methodology blends components of systematic review processes with a streamlined strategy to provide a comprehensive and timely synthesis of the current state of research.

Literature Search: A systematic search of relevant literature was our initial step. We accessed databases, including PubMed, Scopus, and Web of Science, aiming to collect a broad selection of papers discussing the use of drones in healthcare, particularly in emergency services. The search was conducted using a combination of keywords, such as “drone”, “UAV”, “healthcare”, “mobile application”, “system”, “emergency services”, and “GPS location”. The search was confined to English-language literature to ensure consistency in our analysis.

Study Selection: Following the literature search, we applied predetermined inclusion and exclusion criteria to select studies. The criteria for inclusion were original research articles and literature reviews that discussed the use of drones in healthcare directly. Conversely, conference proceedings, editorials, meeting notes, news articles, abstracts, and non-relevant publications were excluded.

Rapid Review and Quality Assessment: After selecting the studies, we conducted a rapid review, an approach that expedites the traditional systematic review process while maintaining the rigor and reliability of the results [15]. The selected articles were evaluated for their relevance and quality, focusing on their methodology, findings, and conclusions. This rapid review approach enabled us to quickly synthesize the current understanding of drone use in healthcare, fulfilling the primary objective of this study.

**Figure 1:**
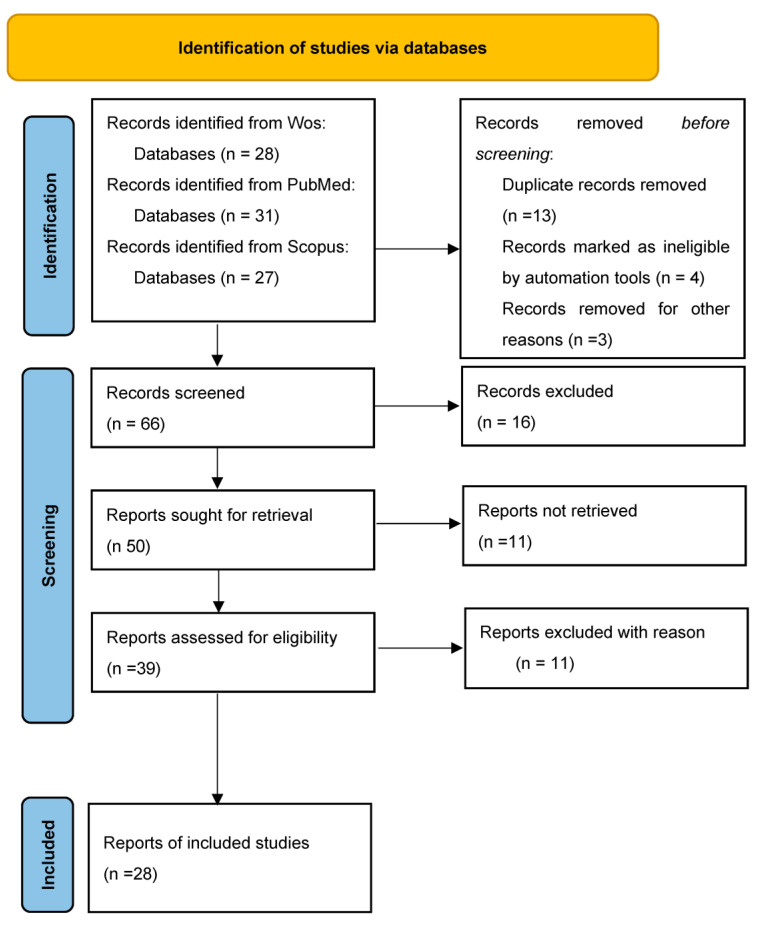
Flow Diagram of Literature Selection Process.

Data Extraction and Synthesis: In parallel with the rapid review process, a content analysis was performed to identify common themes, trends, and discrepancies in the findings, thus enhancing the insights drawn from the study (Figure 1).

Figure 1 shows the process of literature selection according to the PRISMA flow chart.

## 3. Results

A total of 28 studies were ultimately included in our analysis. All relevant information was systematically categorized and subjected to qualitative assessment. Through the application of content analysis, we identified and outlined the following key topics, which are discussed in detail below.

### 3.1. Drones in Disaster Response

The potential of drones in disaster response has gained significant attention in Saudi Arabia due to the country’s vulnerability to disasters, such as floods, sandstorms, and earthquakes. Drones have emerged as a valuable tool in disaster response owing to their ability to efficiently navigate hard-to-reach areas, provide real-time aerial images and videos of affected regions, and deliver essential supplies. This technology has enabled emergency responders to quickly evaluate the extent of damage, assess potential risks, and coordinate their response efforts accordingly. This has been demonstrated in previous instances where drones have been deployed during disasters in Saudi Arabia, such as during flash floods, earthquakes, and other emergencies [16].

By leveraging drone technology, disaster response efforts in Saudi Arabia can be more effective and efficient, saving valuable time and resources in the process [17]. In addition to their role in search and rescue operations, drones can also be employed for damage assessment, monitoring critical infrastructure, and mapping disaster-affected areas. These applications can provide crucial information to decision makers, allowing for a more targeted and well-informed response.

The integration of drones in disaster response in Saudi Arabia has brought up critical concerns related to safety, privacy, and security. The use of drones in densely populated areas can pose potential privacy law violations, and there is always a risk of accidents or malicious use. To address these issues, the government has introduced strict regulations and guidelines for the responsible and safe use of drones in disaster response [18]. These measures are aimed at ensuring that the use of drones in emergency situations does not compromise the safety and privacy of citizens.

In doing so, Saudi Arabia is setting an example for other countries looking to leverage drone technology in disaster response, highlighting the importance of addressing these issues in a proactive and responsible manner. As drone technology continues to evolve and improve, it will be crucial for regulatory frameworks and guidelines to keep pace with the rapidly changing landscape, ensuring that the benefits of drones in disaster response can be harnessed without compromising the safety and well-being of the affected communities.

### 3.2. Drones in Prehospital Care Delivery and Remote Areas

Effective and timely prehospital care delivery is of paramount importance, particularly in remote and hard-to-reach areas in Saudi Arabia [19]. Drones, with their ability to swiftly transport medical supplies, equipment, and personnel to emergency scenes, can play a significant role in enhancing patient outcomes and potentially saving lives [20].

In Saudi Arabia, several initiatives are currently underway to explore the potential of drones in this context. For instance, the Saudi Arabian Ministry of Health, in collaboration with King Abdullah Medical City, has initiated a pilot project that uses drones for the transportation of medical supplies and equipment across different hospitals [21]. The primary aim of this project is to reduce transportation time and costs, thus improving patient care and outcomes [22].

Additionally, the Saudi Arabian Red Crescent Authority has employed drones for transporting medical supplies and equipment to remote areas. This application is particularly crucial during emergencies and disasters when traditional modes of transportation may be impractical or unavailable [23]. Furthermore, the use of drones in prehospital care delivery provides an avenue for the expansion of telemedicine in Saudi Arabia. With the aid of drones, healthcare professionals could diagnose and treat patients remotely using video conferencing and other telecommunication technologies. Such an approach could be a game changer in remote and rural areas where access to healthcare services is often limited [24].

However, similar to their use in disaster response, the integration of drones in prehospital care delivery is not devoid of challenges and concerns. Regulatory and safety considerations must be duly addressed to ensure the safe and responsible deployment of drones [25]. Moreover, the costs associated with the deployment and maintenance of drone technology might pose a substantial barrier to its widespread adoption [26]. It is, therefore, essential to explore cost-effective strategies for integrating drone technology in prehospital care delivery in remote and rural areas of Saudi Arabia.

### 3.3. Regulatory Considerations

The use of drones in disaster response and prehospital care delivery brings forth several ethical, legal, and safety concerns that necessitate thorough consideration and regulation. To ensure their safe and responsible use, Saudi Arabia has implemented a set of regulations and guidelines that govern the use of drones in healthcare [27].

The Saudi General Authority of Civil Aviation (GACA) has been instrumental in establishing a regulatory framework for drone operation within the country. It mandates the registration, training, and certification of drone operators and sets forth specific guidelines pertaining to drone flight paths, airspace restrictions, and operational times [28]. These regulations also address issues such as privacy, safety, and security, aimed at ensuring that drones are used in a responsible and safe manner, while minimizing intrusion and risks to the public [29].

Complementing the GACA’s regulations, the Saudi Arabian Ministry of Health has issued guidelines specifically targeting the use of drones in healthcare, particularly in disaster response and prehospital care delivery. These guidelines provide detailed instructions on the types of medical supplies and equipment that can be transported by drones, the qualifications and training of drone operators, and the procedures for reporting drone-related incidents and accidents [30]. They also delineate the conditions under which drones can be deployed for healthcare purposes, including emergency situations and routine medical supply delivery to remote areas.

Moreover, these guidelines underscore the importance of maintaining data privacy and security when drones are used for telemedicine or patient monitoring, ensuring that the use of drone technology adheres to existing healthcare privacy laws and regulations.

It is important to note that the regulatory environment for drone use in healthcare is continually evolving, reflecting the dynamic nature of the technology itself. As drone technology advances and its applications in healthcare continue to expand, these regulations will need to be updated and refined to ensure that they continue to protect the public while enabling the benefits of drone technology to be fully realized.

### 3.4. Advancing Healthcare with Drone Technology

The rapid advancement of drone technology has paved the way for an array of opportunities to enhance healthcare delivery, particularly in the realms of disaster response and prehospital care. A crucial innovation is the advent of autonomous drones, which are equipped with sophisticated sensors and algorithms. Their ability to navigate complex environments and avoid obstacles without human intervention allows for expedited delivery of medical supplies and equipment in emergency situations, potentially saving lives and resources [31,32].

In addition, the integration of artificial intelligence (AI) with drone technology marks a significant stride forward in healthcare. AI algorithms can analyze data collected by drones in real time, providing emergency responders with critical insights, such as the location of victims, severity of injuries, and the availability of medical supplies and equipment [33]. This real-time analysis can dramatically enhance the effectiveness of drones in healthcare delivery, especially in disaster-stricken situations.

Moreover, drones are playing a pivotal role in the propagation of telemedicine services. By being equipped with high-resolution cameras and medical instruments, drones can aid in diagnosing and treating patients remotely in hard-to-reach areas, thereby enhancing access to healthcare and curbing transportation costs [34]. This is particularly pertinent in remote and rural areas of Saudi Arabia, where access to medical facilities may be challenging.

However, the swift pace of technological advancements in drone technology is not without challenges. High costs associated with advanced drones and the need for skilled operators to manage these systems are some of the issues that need to be addressed [35]. Furthermore, the implementation of AI and autonomous technology raises additional questions related to ethics, privacy, and data security, which need to be considered and addressed.

Despite these challenges, the fusion of drone technology with emerging technologies, such as autonomous systems, AI, and telemedicine, holds immense promise. These advancements are poised to substantially enhance the effectiveness and efficiency of healthcare delivery in Saudi Arabia and potentially around the globe.

### 3.5. Future Directions

As the evolution of healthcare delivery in Saudi Arabia continues apace, drone technology is set to play a pivotal role in shaping its trajectory. Besides the significant opportunities discussed in preceding sections, there are several other areas where drone technology is revolutionizing healthcare delivery. 

One emerging area of significance is the use of drones in emergency medical services. Owing to their ability to swiftly reach remote or challenging locations, drones have the potential to deliver critical care and transport patients to healthcare facilities promptly [36]. This rapid response can enhance patient outcomes and reduce mortality rates in emergency scenarios.

A further area of innovation lies in the development of drones specifically designed for medical transport [37]. These drones can be fitted with vital medical equipment, such as defibrillators, and can be employed to transport critically ill patients during emergencies. This can significantly improve access to medical care and cut down on the time taken to transport patients who require urgent medical attention.

Telehealth, aided by drones, is another burgeoning area of interest [38]. Leveraging real-time video and data transmission capabilities, drones can bridge the gap between healthcare providers and patients in remote or underserved locations. This can not only improve access to medical care, but also reduce transportation costs and enhance the overall efficiency of healthcare delivery.

Lastly, the advancement of drones with sophisticated sensing capabilities is a promising area of innovation [39]. Drones can be equipped with sensors capable of detecting and monitoring various health parameters, such as vital signs, in real time. This information can be relayed to healthcare providers, enabling them to make informed decisions about patient care, even in remote or underserved locations.

The potential of drone technology in transforming healthcare delivery in Saudi Arabia is vast, with myriad possibilities still unexplored. As this technology continues to evolve and mature, it is poised to play an increasingly significant role in shaping the future of healthcare delivery in the country.

## 4. Discussion

The integration of drones in healthcare delivery has seen accelerated advancement in recent years, transforming the landscape of medical emergency responses, especially in regions such as Saudi Arabia, where topographical and logistical challenges may hinder efficient healthcare provision [40]. This discussion evaluates the potential of drones in disaster response and prehospital care, the regulatory landscape, technological advancements, and future directions for drone usage in healthcare.

The utilization of drones for disaster response and prehospital care is a paradigm shift in healthcare delivery, with substantial potential to augment the current systems in Saudi Arabia. Real-time aerial imagery can expedite situational assessment, enabling more precise deployment of emergency resources. Additionally, drones can transport medical supplies to disaster-stricken or remote areas, thereby improving the response time and potentially increasing survival rates [41]. Yet, the implementation of drone technology in healthcare raises inevitable regulatory and safety concerns. Effective policies and guidelines need to be established to regulate drone usage and ensure public safety [42].

The potential of drones for disaster response is widely discussed in recent literature. For example, Daud et al. highlighted drone applications in emergency management, emphasizing the progressive initiatives by the Saudi Red Crescent Authority [23]. Similar advancements have been made in traffic monitoring and civil defense [43]. These examples underscore the promise of drones in improving disaster response and mitigating the impact of emergencies. Drones can enhance situational awareness, decrease response times, and save resources [44,45].

The integration of drone technology in healthcare is not just limited to disaster response. Recent advancements, such as autonomous drones, artificial intelligence, and telemedicine, have opened up new avenues for healthcare delivery. For instance, autonomous drones equipped with AI can improve the efficiency of routine tasks, such as delivering medical supplies or capturing images for disease diagnosis [46]. Furthermore, telemedicine applications can enable remote patient monitoring and consultation, reducing the burden on healthcare facilities [47].

However, the path to integrating drones into the healthcare system is fraught with challenges. These include the need for skilled operators, high acquisition and maintenance costs, and the absence of a supporting infrastructure [48]. Furthermore, the current national healthcare policy and strategic health sector transformation plan do not adequately address the use of drones in prehospital emergency care and disaster medicine [49,50].

The integration of drone technology into the Health Sector Transformation Program, aimed at restructuring Saudi Arabia’s health sector by 2030, is an essential next step. This should be a focal point of the initiative, given the technology’s potential to revolutionize healthcare delivery [51].

The NEOM project, based in the northwestern part of Saudi Arabia, is an endeavor that plans to establish a smart city equipped with an advanced health ecosystem. The project provides a platform for the exploration and implementation of drone technology, which has the potential to significantly alter healthcare delivery within the city [52].

The project is fundamentally anchored in technological innovation, and drone technology is a key part of this vision. Drones have the potential to enhance the city’s healthcare system in several ways: They can enable the rapid transportation of medical supplies, support real-time monitoring of health emergencies, and assist in the provision of telemedical services. In addition, drones could contribute to the mapping and surveying of the city’s infrastructure, thereby helping to optimize the placement and distribution of healthcare facilities [52].

A notable development within the NEOM project is a partnership with Volocopter, a company specializing in urban air mobility. This collaboration seeks to develop a three-dimensional public health transportation system, which could redefine how healthcare services are delivered [52]. This system may include drones designed to transport medical professionals and critical care patients, potentially reducing transportation times during emergencies. Moreover, these drones could be used for routine medical supply deliveries, connecting city-based healthcare facilities with those in remote or hard-to-reach areas.

The partnership with Volocopter highlights the potential role of drone technology in modifying healthcare delivery. However, realizing this potential will require the establishment of a comprehensive regulatory framework to ensure the safe and efficient operation of drones. The NEOM project thus serves as a potential model for the future integration of drone technology in healthcare. If successful, it could provide an example of how drones can be effectively utilized within a city’s healthcare ecosystem, addressing logistical challenges and enhancing patient care [52].

The applications of drones in healthcare extend far and wide, ranging from critical disaster response to the regular delivery of healthcare services. Their ability to swiftly reach areas otherwise inaccessible by traditional transportation means, coupled with their potential to support telemedical services, underscores the broad-ranging impact they could have on healthcare delivery.

Saudi Arabia, with its continued commitment to technological innovation and investment in drone technology, is well positioned to spearhead this transformative wave in healthcare. The nation’s strategic focus on drone technology, as evidenced in initiatives such as the NEOM project and Medidrone system, demonstrates its forward-thinking approach and readiness to embrace this advancement [53,54].

Saudi Arabia’s potential to emerge as a global leader in healthcare drone technology is not merely a theoretical aspiration. The country’s ongoing advancements in drone technology, combined with its unique geographical and logistical challenges that can be addressed by drones, present a substantial opportunity for Saudi Arabia to lead the field and set international precedents. However, the path towards broad-scale implementation of drones in healthcare is not without significant hurdles. These include, but are not limited to, the need for an adequately trained workforce capable of operating and maintaining advanced drone systems, addressing privacy and safety concerns, and managing the high upfront costs associated with procuring and maintaining drone fleets.

Another critical element in this journey is the creation of a conducive regulatory environment. Regulatory frameworks need to be carefully designed to ensure the safe and ethical use of drones, respecting privacy laws and securing data transmission. Collaborative efforts involving policymakers, healthcare professionals, drone manufacturers, and other stakeholders will be essential in establishing these regulatory frameworks, ensuring that they support innovation, while prioritizing safety and privacy [53,54].

The potential of drones in transforming healthcare delivery is vast, and with a strategic focus on technological innovation, robust investment, and a well-designed regulatory environment, Saudi Arabia could take a global leadership role in this domain. The challenges to be overcome are considerable, but the potential benefits for patient care and healthcare efficiency make this a journey worth undertaking.

## 5. Limitations

The limitations of the current study should be taken into account while interpreting the findings.

Firstly, the scope of the content analysis was confined to the available rapid review literature, which may have been subject to selection bias. This study focused on articles available in online databases and published in English, potentially overlooking relevant studies published in other languages or other types of sources. Thus, the results may not be representative of the full spectrum of research on the topic, which could limit the generalizability of the findings.

Secondly, personal and professional biases of the researcher could have influenced the inclusion and exclusion criteria, potentially introducing bias into the content analysis. To mitigate this, future studies could employ multiple reviewers or use systematic and objective methods for article selection.

Thirdly, the quality of the included studies may have varied, which could have affected the overall reliability and validity of the findings. Future research should include a comprehensive quality assessment of the studies included in the analysis to enhance the robustness of the findings.

In addition to these study-specific limitations, the implementation of drone technology in healthcare delivery presents not only opportunities, but also significant challenges. A crucial challenge is the issue of safety. The drones need to operate in a variety of weather conditions, often in complex and busy urban environments. This brings the risk of collisions, not just with other drones, but also with buildings, people, or other aircraft.

Regulatory hurdles are another significant limitation to drone usage in healthcare. Given the variety of potential uses for drones in healthcare, a one-size-fits-all regulatory approach may not be sufficient. Ensuring the privacy and security of data captured by drones, particularly given the sensitive nature of health-related information, is crucial.

Technological constraints are also of concern, with issues such as drone payload capacity, range, and endurance, as well as their reliability in varied environments, posing significant challenges. From an operational perspective, drones require skilled operators, a challenge that developments in autonomous drone technology may alleviate, but not entirely eliminate.

Finally, limitations associated with drone usage in emergency or disaster situations should also be acknowledged. For instance, connectivity issues and limitations of drone power systems can hinder their effective deployment in disaster zones. Moreover, the disruption of different infrastructures, including communication and network systems after earthquakes or other disaster types, can further compromise the functionality of drones.

Despite these limitations, this content analysis provides valuable insights into the most important topics for the integration of drones in healthcare delivery in Saudi Arabia. Future research should aim to address these limitations and explore more in-depth how drone technology can be used to improve health outcomes, reduce costs, and improve the efficiency of prehospital care delivery in remote and rural parts of the country.

## 6. Conclusions

The potential of integrating drones into healthcare delivery in Saudi Arabia is substantial, with promising implications for improving patient outcomes, particularly in disaster response and prehospital care. This content analysis of rapid review literature has illuminated several key discussion points, including the application of drones in disaster response and prehospital care, regulatory considerations, technological advancements, and future trajectories for drone usage in healthcare.

There exist challenges and concerns that need to be addressed, such as the requirements for trained operators, connectivity issues, and the high costs associated with drone acquisition and maintenance. However, the potential benefits of improved healthcare access, reduced transportation time and costs, and enhanced patient outcomes underscore the significant potential of drone integration in Saudi Arabia’s healthcare sector.

Regulatory and safety considerations are paramount for the safe and responsible use of drones in healthcare. This necessitates ongoing efforts to address these concerns and to stimulate the development and deployment of advanced drone technology. It is also critical to ensure that drone usage is included in the national healthcare policy framework and the strategic health sector transformation plan.

Additionally, technological advancements, such as the development of autonomous drones and the integration of artificial intelligence, could further enhance the effectiveness and efficiency of drone usage in healthcare delivery, particularly in areas with limited access to healthcare services.

Moreover, initiatives such as NEOM, which aim to create a digital smart city with an integrated health ecosystem based on advanced technology, could provide a fertile ground for drone technology experimentation and research, paving the way for widespread implementation in the healthcare system.

By actively engaging with stakeholders and leveraging emerging technologies, Saudi Arabia can tap into the full potential of drones in healthcare delivery, ensuring that the country remains at the cutting edge of healthcare innovation and technology.

In conclusion, despite the challenges, the endless possibilities for drone use in disaster medicine support and prehospital care delivery underscore the importance of continued innovation and investment in drone technology, with Saudi Arabia poised to lead the development of drone use in these fields.

## Data Availability

The datasets used and/or analyzed during the current study are available from the corresponding author on reasonable request.
